# Role of the Vitreous in Retinal Pathology: A Narrative Review

**DOI:** 10.7759/cureus.43990

**Published:** 2023-08-23

**Authors:** Stella-Ioana Popescu, Mihnea Munteanu, Cristina Patoni, Andreea Mihaela Alexandra Musat, Vlad Dragoescu, Corina-Cristina Cernat, Marius-Nicolae Popescu, Ovidiu Musat

**Affiliations:** 1 Ophthalmology, Central Military Emergency University Hospital “Dr. Carol Davila”, Bucharest, ROU; 2 Ophthalmology, University of Medicine and Pharmacy “Victor Babeş”, Timisoara, ROU; 3 Ophthalmology, University of Medicine and Pharmacy "Victor Babeş”, Timisoara, ROU; 4 Gastroenterology, University of Medicine and Pharmacy "Carol Davila", Bucharest, ROU; 5 Gastroenterology, Central Military Emergency University Hospital “Dr. Carol Davila”, Bucharest, ROU; 6 Ophthalmology, University of Medicine and Pharmacy "Carol Davila", Bucharest, ROU; 7 Ophthalmology, Central Military Emergency University Hospital "Dr. Carol Davila", Bucharest, ROU; 8 Physical and Rehabilitation Medicine, Elias Emergency University Hospital, Bucharest, ROU; 9 Physical Medicine and Rehabilitation, University of Medicine and Pharmacy "Carol Davila", Bucharest, ROU

**Keywords:** diabetic retinopathy, vitreomacular traction, vitreoretinal interface, posterior vitreous detachment, vitreous body

## Abstract

The vitreous body is an anatomically and biochemically complex structure. Because of its proximity and firm adherence to the retina, researchers have examined the link between these two structures and how their individual pathologies might be connected. Several experimental and clinical studies have already demonstrated the important role of vitreous in the pathogenesis of retinal disorders.

This narrative review highlights the role of the vitreous in retinal diseases and the improvements that have been made since the introduction of optical coherence tomography. This leads to a better understanding of vitreoretinal diseases and demonstrates its determinant role in other retinal pathologies, such as diabetic retinopathy or age-related macular degeneration.

As we deepen our knowledge of the vitreous's structure, function, and abnormal conditions, we can better link the changes in diseases and identify effective treatments.

## Introduction and background

The vitreous body is a gelatinous structure that occupies approximately four-fifths of the volume of the eye. Its volume is about 4 mL, weighing approximately 4 g. It is attached through the hyaloid, the external membrane of the vitreous body, on the entire retinal surface. This attachment is stronger in certain regions, such as the vitreous base, the disc margin, the perifoveal region, the retinal vessels, and the periphery of the posterior lens capsule. We thus refer to the vitreous base, the region at which the vitreous attachment is the strongest. The vitreous base is the circumferential region corresponding to the posterior 2 mm of the pars plana, to the 1 to 4 mm of the retina posterior to the ora serrata. It is composed of 99% water, 0.9% being occupied by salts and low molecular weight lipids, and 0.1% occupied by proteins (collagen) and hyaluronic acid. Hyaluronic acid is a molecule that, due to its weak negative electrical charge at physiologic pH, is hydrophilic. Thus, it is responsible for the state of hydration of the vitreous body, as well as its viscosity. The main types of collagen found in the vitreous body are collagen type II and collagen type IX, which give elasticity to the vitreous. In the case of young vitreous, hyaluronic acid and collagen are distributed uniformly, and therefore the vitreous has a homogeneous character. The cells at the level of the vitreous body are rare and are mainly represented by hyalocytes located at the level of the hyaloid membrane [1,2].

A consequence of vitreous degeneration is vitreous shrinkage and posterior vitreous detachment (PVD). These processes, in turn, may lead to the development of vitreomacular interface disorders if the vitreomacular adherence is too strong and traction becomes significant, and to rhegmatogenous retinal detachment if traction causes a retinal break in the periphery. Other, more subtle functions of the vitreous in retinal pathology would be that of a reservoir for different molecules, such as cytokines. This is thought to be of great importance in diabetic retinopathy (DR) pathogenesis.

Vitreous pathogenesis is becoming increasingly important with the expansion of vitreoretinal surgery. This article provides a brief overview of what is currently known about the vitreous, with a focus on its metabolic function. We review the pathogenesis of vitreomacular interface disorders (vitreomacular traction, macular hole, and epimacular membrane), rhegmatogenous and tractional retinal detachment, DR, and macular edema. In addition, pure vitreal pathologies associated with potential visual disturbance, such as asteroid hyalosis, synchysis scintillans, and vitreous amyloidosis, are discussed.

## Review

Degenerative changes of the vitreous body: asteroid hyalosis, synchysis scintillans, and amyloidosis

Asteroid hyalosis (AH) is characterized by bright yellowish-white particles surrounded by tightly adherent fibers inside the vitreous cavity [3]. AH is a relatively common cause of vitreous opacities, possibly associated with diabetes mellitus (DM) and hereditary pigmentary retinopathies [4]. Although some individuals experience visual discomfort or blurring, especially if the opacities are densely crowded close to the macula, AH is generally asymptomatic in most patients [5]. The etiology of AH remains to be elucidated. It has been suggested that releasing phospholipids and calcium from degenerating retinal cells could produce AH. Biochemical methods, and optical and electron microscopy were used to study the structure and composition of asteroid bodies. Phospholipids and calcium are proposed to be their major components. Systemic diseases such as DM, systemic hypertension, and hyperlipidemia have been associated with AH. Hypercholesterolemia, elevated serum calcium levels, and gout are also described in small studies as being associated with AH [3]. Synchysis scintillans is an uncommon eye disorder that occurs when cholesterol crystals gather in the vitreous. Cholesterol crystals appear as small, refractive opacities that move freely in a gravity-dependent manner. Even if it is commonly found in eyes affected by other pathologies, synchysis scintillans is often asymptomatic and discovered by chance. The opacities observed in synchysis scintillans were demonstrated by chromatography as pure cholesterol. In synchysis scintillans, cholesterol crystals are distinguishable from asteroid bodies through their larger size and tendency to settle at the bottom. The risk factors for synchysis scintillans include DR, vitreous hemorrhage, chronic uveitis, ocular trauma, retinal detachment, hypermature cataract, and Coats disease. There may be several reasons for the occurrence of synchysis scintillans, a condition characterized by the presence of mobile cholesterol crystals in the vitreous humor of the eye. In some cases, it could be due to the release of cholesterol from broken red blood cells, which may explain instances associated with vitreous hemorrhage. However, this explanation cannot account for cases that do not involve hemorrhage, such as retinal detachment or hypermature cataracts. In the case of retinal detachment, cholesterol is believed to originate from subretinal fluid that passes through retinal tears and diffuses into the vitreous humor [6]. In cases of hypermature cataracts, the presence of exudative lens material can cause the formation of cholesterol crystals within the eye. Finally, intraocular inflammation has been proposed as a pathogenic mechanism. A recent study suggests that oxidative stress and lipoperoxidation can cause the formation of intraocular cholesterol crystals [6].

Amyloidosis refers to a range of diseases that are characterized by the accumulation of hyaline extracellular material in different tissues of the body, including the eyes and their appendages. This deposition occurs due to the abnormal folding of amyloid proteins, which then form insoluble fibrils. Diagnosis is confirmed through Congo red dye affinity and green birefringence under polarized light microscopy [7]. Involvement of all ocular compartments has been reported, including the adnexa, extrinsic muscles, levator palpebrae, conjunctiva, cornea, lens, uvea, and trabecular meshwork, as well as the vitreous and retina. Vitreous amyloidosis is classically described as initially granular, with deposits increasing in size and aggregating to form a "glass wool appearance." Deposits are usually bilateral, although they can be asymmetric or unilateral. Vitreous involvement is commonly seen in systemic amyloidosis, but there have also been cases of isolated ocular disease [7]. The occurrence of cloudy areas in the vitreous due to hereditary amyloid transthyretin (ATTRv) amyloidosis can range from 5.4% to 35%. Collagen fibers in the vitreous play a role in the buildup of transthyretin (TTR) amyloid. TTR protein has a high affinity for basement membranes, and vitreous matrix is predominantly composed of type II collagen, which has structural and biochemical similarities to collagen in basement membranes. The current standard treatment is surgical with a 25-gauge pars plana vitrectomy (PPV) [8].

Natural changes: posterior vitreous detachment

With advancing age, the vitreous undergoes a series of degenerative changes called synchysis or liquefaction, respectively, syneresis. The vitreous liquefies when hyaluronic acid molecules redistribute and cause a subsequent redistribution of water molecules. Thus, pockets or lacunae of liquefied vitreous are formed, and, in the outstanding regions, collagen molecules will aggregate. This molecular redistribution is responsible for the decrease in the volume of the vitreous body, a process called syneresis. Risk factors for early vitreous degeneration include aphakia, pseudophakia, myopia, female gender, menopause, eye inflammation and trauma, conditions such as retinitis pigmentosa and Stickler syndrome, and invasive interventions on the eye (eye surgery, laser treatment, or cryotherapy). The attachment of the hyaloid membrane to the internal limiting membrane (ILM) also weakens typically with age. PVD occurs as a result of certain predisposing factors. In order for this to happen, there must be a degeneration of the vitreous, as well as a weakening of the vitreoretinal adhesions. Liquefied vitreous penetrates through defects at the level of the hyaloid into the space between the hyaloid and the ILM, creating a plane of dissection between these two structures. The prevalence of PVD increases with age [1,2,9]. It is common for patients over 45 years old to experience a spontaneous PVD due to aging. In patients aged 50-59 years, approximately 24% experience this condition, which increases to around 87% for those in the age group of 80-90 years [10].

PVD begins in the perifoveal region, later moving anteriorly and stopping at the vitreous base. At this level, the vitreous will remain attached. In the case of a physiological PVD, associated with age, it is usually slow and asymptomatic. The final stage, involving the rim of the optic disc, is usually symptomatic due to the appearance of the Weiss ring [1,2,11]. When symptoms do develop, the main complaints of patients with PVD are flashes, floaters, and/or reduced vision. PVD is associated with an increased risk of developing retinal tears, retinal detachment, and vitreous hemorrhage, and, thus, patients experiencing these symptoms are usually directed to the emergency room of the ophthalmology department [12].

Role of the vitreous in vitreoretinal interface disorders

The adhesion mechanics at the vitreoretinal interface (VRI) play a significant role in the development of several illnesses that result in visual impairment or blindness. When there are strong focal vitreoretinal adhesions, it can impede complete PVD. Such adhesions can apply tension on the retina, causing macular holes, and epiretinal membranes (ERMs) in the macula [13].

The process of PVD can become pathological when vitreous liquefaction occurs without concomitant weakening of the VRI. Only partial separation of the vitreous from the retina is achieved. This condition is called abnormal PVD or incomplete or partial PVD. The same risk factors for early vitreous degeneration may be responsible for these syndromes, as they decrease vitreous volume at an age when vitreoretinal adhesions are still strong.

When the area where the attachment persists is the macula, we speak of vitreomacular adhesion. Traction forces associated with the collapse of the vitreous body at the vitreomacular adhesion give rise to vitreomacular traction syndrome. It can progress to the mechanical interruption of the retinal layers, a phenomenon called retinoschisis, or to the avulsion of a portion of the retinal tissue, which leads to the formation of a macular hole [14-17].

A stage 0 hole is a type of eye condition where a PVD occurs but the fovea (a small depression in the retina) remains attached. This can be detected through a slight change in the foveal depression. Generally, the level of visual acuity is not impacted. It is important to note that most stage 0 holes do not progress to more advanced stages.

When someone has a stage 1 macular hole, they may experience visual symptoms such as metamorphopsia and a decline in central vision. Their visual acuity range may be between 20/25 and 20/60. The distinctive signs of this condition are either a small yellow spot (known as stage 1A) or a yellow circle (known as stage 1B) located in the fovea. After conducting an optic coherence tomography (OCT) examination, it was discovered that in stage 1A, there is a foveal "pseudocyst" or horizontal split (cleft) linked to vitreofoveal traction. When the hole is in stage 1B, there is a lack of substance in the outer fovea, which results in a yellow ring visible during clinical observation. It is worth noting that around 50% of cases of stage 1 holes may resolve spontaneously.

At stage 2 of macular hole, there is a hole in the center of your retina that is less than 400 μm in diameter. This happens when a split in the retina grows and becomes a full hole. As the hole grows, your vision may become worse. An OCT scan can show the full hole and the attachment of a part of your eye called the posterior hyaloid to the center of the hole [2].

A stage 3 macular hole is a hole greater than 400 μm in diameter. The posterior hyaloid remains attached at the optic nerve level, but is detached from the fovea. An operculum suspended by the posterior hyaloid can be seen. On OCT, this stage represents a large macular hole without vitreo macular traction (VMT).

A stage 4 macular hole is a fully developed hole with a complete PVD, evidenced by the presence of a Weiss ring. On OCT, this stage also represents a large macular hole without VMT [2,16].

Macular hole surgery has come a long way since the time when it was considered untreatable. It is now considered one of the most successful surgeries. One essential step of this surgery is ILM peeling, which has been established as a crucial component in the etiopathogenesis and progression of macular hole [18]. Other successful surgical techniques used when ILM peeling fails are ILM flap creation and closing the hole with human amniotic membrane, lens capsule, and autologous platelet concentrate [19].

The pathogenesis of epimacular membranes needs to be completed and elucidated. Most of them are classified as idiopathic ERMs [20,21]. The prevalence of ERM is 7% to 11.8%, with increasing age being the most important risk factor [22]. Fibrocellular proliferation over the ILM is characteristic of an ERM. The initial event is a PVD. PVD causes dehiscences in the ILM, allowing microglial cells to migrate to the retinal surface, interacting with hyalocytes and laminocytes at this level. These cells later differentiate into fibroblast-like cells to form a thin cellophane-like ERM. A prerequisite for forming the epimacular membrane is, therefore, the phenomenon of PVD. The following three theories have been proposed for the pathogenesis of epimacular membranes. First, microglial cells originating in the inner neurosensory retina migrate to the surface of the inner limiting membrane through defects produced at its level following a retinal tear or following PVD. These cells later differentiate into fibroblasts that form the epimacular membrane. The second one is that after PVD, segments of cellular vitreous remain on the surface of the ILM. These segments contain hyalocytes that differentiate into myofibroblasts that form the epimacular membrane. The third one refers to PVD-induced ILM avulsion in the posterior paravascular retina stimulates cytokine production that leads to ERM production [20,21].

Vitrectomy with membrane peeling remains the mainstay of treatment for symptomatic ERMs [22].

Role of the vitreous in macular edema

Starling's law describes the formation of edema in tissues. It states that hydrostatic and osmotic forces control fluid transport between tissue and blood vessels. The difference in hydrostatic pressure between blood vessels and tissue drives water into the interstitial space. The difference in osmotic pressure absorbs water from the tissue into the blood vessels due to the high osmotic activity of the macromolecules in the blood. In usual circumstances, there is a balance between the hydrostatic and osmotic pressure in tissues and blood vessels, preventing any water movement. However, when there is vitreomacular traction, the pressure in the retina decreases, causing an increase in the pressure difference between the blood vessels and retinal tissue. This upsets the equilibrium and leads to water transport. Water thus accumulates in the tissue in the form of edema. The water accumulation lowers the osmotic pressure in the retinal interstitial space and increases the osmotic pressure difference until a new equilibrium is established between the hydrostatic and osmotic pressure differences. At this stage, there is no net transportation of water and the tissue reaches a state of equilibrium, resulting in macular edema. It is suspected that the lack of a complete PVD could be a risk factor for developing particular forms of macular edema, such as diabetic macular edema (DME) or Irvine-Gass syndrome [23].

Role of the vitreous in diabetic retinopathy

DR is a frequent microvascular complication of DM and is responsible for most vision loss in older individuals. Elevated blood sugar and changes in metabolic processes result in oxidative stress and neurodegeneration in the early stages of DR. The duration of DM, high blood sugar levels, and hypertension are closely linked to the development of DR. A higher level of HbA1c is significantly linked to the advancement of DR [24,25]. Poor glycemic control, systemic hypertension, DM duration, dyslipidemia, microalbuminuria, and local inflammation are certain factors that pose a significant risk for the development and progression of DR. Recent studies have identified that higher aortic stiffness can indicate the likelihood of developing DR and peripheral neuropathy [26].

When blood vessels in the retina are exposed to high levels of glucose, they initially respond by dilating and changing blood flow. Additionally, the loss of pericytes - cells responsible for providing structural support to capillaries - is a key feature of early DR [27]. In both in vivo and in vitro studies, it has been shown that pericytes undergo apoptosis, or programmed cell death, when exposed to high glucose levels. Without pericytes, the capillary walls can bulge out, leading to the formation of microaneurysms, which are the first signs of DR [28,29].

When pericytes and endothelial cells are significantly lost, it causes capillaries to be blocked and leads to ischemia. In the retina, this ischemia or lack of oxygen causes an increase in vascular endothelial growth factor (VEGF) due to HIF-1 activation [30]. The primary factor that determines angiogenesis and cell growth in DR is the balance between VEGF and angiogenic inhibitors [31].

Moreover, the duration and severity of DM can impact the vitreous body's structure in two ways. Firstly, it increases the likelihood of collagen glycation and crosslinking of collagen fibrils with other structural proteins [32]. Secondly, it can affect the posterior VRI's structure. Changes in the VRI can lead to the development of an ERM, which is a significant indicator of proliferative diabetic retinopathy (PDR) [33,34]. Fibrovascular membranes in the vitreous can lead to retinal hemorrhages, detachment, and eventual loss of vision.

Treatment options remain dramatically limited. Over the past decade, intravitreal anti-VEGF agents have become the first-line therapy for DME and PDR. Laser photocoagulation still plays an important role in the treatment of DR as an adjuvant treatment [35]. If the anti-VEGF treatment and laser are combined, the number of injections could be reduced [36].

Role of the vitreous in rhegmatogenous retinal detachment

Retinal detachment represents the presence of liquid between the retinal pigment epithelium (RPE) layer and the neurosensory retina. In rhegmatogenous detachment, the fluid reaches the subretinal space through a retinal hole or tear. The origin of the subretinal fluid is the liquefied fraction of the vitreous body, and, thus, it depends on synchysis, while the retinal tear is most often associated with the phenomenon of PVD. An abnormally tight focal adhesion associated with consecutive vitreous syneresis traction causes these retinal tears. They are most often found along the posterior margin of the vitreous and in regions of degenerative retinal thinning called lattice degeneration.

To cause a rhegmatogenous retinal detachment (RRD), the following three factors must be present: liquefied vitreous gel, traction forces that can cause a retinal tear, and a retinal tear that allows liquefied vitreous to pass into the subretinal space. These are the defining characteristics of an RRD [2,37].

RRD, which was previously considered untreatable, now has primary surgical success rates of more than 80% to 90%. Even complex cases are now treatable. There is a lot of debate about the best way to manage RRD. Surgeons differ in their preferences based on their experience and the types of cases they handle, as well as the availability of equipment. The main options are pneumatic retinopexy, scleral buckling, and vitrectomy. The primary goal of managing RRD is to reattach the retina. While the benefits of treating asymptomatic or chronic RRD are uncertain, surgery is strongly recommended for symptomatic cases. RRD is typically categorized into two types: "macula-on," where the foveal center is not attached, and "macula-off," where the fovea is detached. Individuals with macula-on RRD typically have better initial visual acuity and a more favorable prognosis with successful surgery. Macula-off RRDs have lower initial best-corrected visual acuity and worse visual prognosis, even with successful retina reattachment [37]. Prompt surgery for macula-off RRD enables the gradual restoration of the outer retinal layers, resulting in significant improvement in visual function, according to research [38].

Role of the vitreous in tractional retinal detachment

Tractional retinal detachment (TRD) happens when the neurosensory retina separates from the RPE due to tractional forces caused by proliferative membranes located on the retina or vitreous surface. These membranes can develop due to different types of proliferative retinopathies, with the most common being PDR [39].

In cases of PDR, the pathophysiology of TRD has been well described. Chronic hyperglycemia causes capillary obstruction and ischemia, resulting in increased levels of nitric oxide (NO). This, in turn, increases activity of protein kinase C and activates vascular growth factors, such as VEGF and several chemokines. New blood vessels can form due to specific growth factors. These vessels can penetrate the ILM and grow into the vitreous cavity. The posterior hyaloid of the vitreous serves as a framework for the growth of these new vessels. If the posterior vitreous completely detaches in early stages of DR, it can decrease the risk of progression to PDR [40].

Later, glial cells engulf the neovessels, leading to the formation of fibrous tissue. Over time, this tissue made of fibers contracts and pulls on the surface of the retina. This can cause the neurosensory retina to detach from the RPE. Alternatively, adhesion between the retina and posterior vitreous provided by fibrovascular tissue associated with vitreous syneresis will lead to traction and tractional retina detachment. The pathophysiology of TRD in other proliferative retinopathies can be explained similarly to that in PDR [39].

Vitrectomy surgery is indicated for recent less than six months’ duration) TRD involving the macula and progressive TRD that threatens the macula, and recent data suggest that chronic macula-involving TRDs (more than six months’ duration) may also benefit from surgery [41].

Myopia and vitreous changes

Myopia, also known as short-sightedness, is a refractive error where light from far distances is focused in front of the retina [42]. Pathological myopia, also known as degenerative or progressive myopia, is often accompanied by degenerative changes in the eye. Severe myopia can cause atrophy of the outer retina, the RPE, and the choroid, as well as cracks in Bruch's membrane and thinning of the sclera. A chronic shallow serous detachment of the macula can occur in some myopic patients with macular and juxtapapillary staphylomas, even without other associated conditions. The cause of this type of retinal detachment is uncertain, but it may be caused by vitreous traction in the presence of a staphyloma. High-resolution OCT can distinguish between a macular schisis, a retinal detachment, or a combination of both [43].

Retinal tears occur due to abnormal vitreoretinal adhesions, which cause the detaching posterior vitreous to pull and create a tear in the normal retinal tissue [44]. This can result in either a flap of retinal tissue remaining attached or a completely avulsed retinal fragment. PVD is the cause of symptomatic tears, while asymptomatic tiny tears with either an attached flap or a free operculum are usually caused by cystic retinal tufts. On the other hand, atrophic holes develop due to a gradual thinning of the retinal tissue within lesions of lattice degeneration.

Imagistic in vitreoretinal diseases

Ultrasonography is a diagnostic tool used to assess the density of the vitreous, confirm retinal attachment, and detect any swelling in the choroid [45]. Ultrasonographically, PVD appears as a membranous, linear, fine, concave echo with undulating movements that appear after the movement of the eyeball [46]. The opacity may or may not be attached to the optic nerve head, as the PVD is complete or incomplete. The other vitreoretinal diseases have a similar appearance on ultrasound: multiple, punctate, inhomogeneous echoes with medium to large reflectivity scattered throughout the vitreous due to pathological materials such as calcium-lipid complexes (AH), cholesterol crystals (synchisis scintillans), and protein materials (amyloidosis).

The OCT is a revolutionary tool that has greatly improved the diagnosis of macular disease. Using OCT evaluation, we can see a tractional elevation of the retina, which leads to intraretinal fluid accumulation, retinal thickening, and subretinal fluid accumulation [47]. An OCT scan can also detect a full-thickness defect in the neural retina, distinguishing a real macular hole from a pseudo hole that appears during a clinical examination [48]. When evaluating patients with DR, OCT can provide crucial information about the retina thickness and the retinal edema level. In patients with DME, a common finding is an intraretinal focal hyperreflective that clinically corresponds to retinal exudates. Additionally, OCT scans can detect focal vitreoretinal adhesions not visible during clinical examinations, which can help determine whether vitrectomy and membrane peeling are necessary.

## Conclusions

Degenerative changes in the vitreous body and its relationship with the retinal surface have a primary role in the occurrence of many retinal diseases. The presence of abnormal PVD is crucial in the development of vitreomacular interface disorders and RRD. Unusually strong vitreomacular adherence is a key feature in vitreomacular traction and macular hole pathogenesis, while peripheral adherence commonly leads to RRD. Hyalocytes and the ILM integrity might be essential factors in epimacular membrane occurrence. Longtime unknown, the role of the vitreous in DR and macular edema became another area of intense research.

Historically, gaining insight into RRD pathogenesis (vitreous traction, adherence, tear formation) led to logical interventions that aimed to release traction and seal tears. Similarly, advances in imaging technologies and the arrival of OCT in clinical practice and research solved many of the vitreomacular interface disorders’ mysteries and revolutionized treatments and research. We can suppose that by continuously studying vitreoretinal pathophysiology, we could answer other disease mechanisms and, by doing so, develop novel therapies.
